# Shift in Demographic Involvement and Clinical Characteristics of COVID-19 From Wild-Type SARS-CoV-2 to the Delta Variant in the Indian Population: In Silico Analysis

**DOI:** 10.2196/44492

**Published:** 2024-10-08

**Authors:** Ashutosh Kumar, Adil Asghar, Khursheed Raza, Ravi K Narayan, Rakesh K Jha, Abhigyan Satyam, Gopichand Kumar, Prakhar Dwivedi, Chetan Sahni, Chiman Kumari, Maheswari Kulandhasamy, Rohini Motwani, Gurjot Kaur, Hare Krishna, Sujeet Kumar, Kishore Sesham, Sada N Pandey, Rakesh Parashar, Kamla Kant

**Affiliations:** 1 Department of Anatomy All India Institute of Medical Sciences-Patna Patna India; 2 Department of Anatomy All India Institute of Medical Sciences-Deoghar Deoghar, Jharkhand India; 3 Department of Anatomy All India Institute of Medical Sciences-Bhubaneshwar Bhubaneshwar India; 4 Department of Anatomy All India Institute of Medical Sciences-Gorakhpur Gorakhpur India; 5 Department of Anatomy Postgraduate Institute of Medical Education and Research Chandigarh India; 6 Department of Biochemistry All India Institute of Medical Sciences-Madurai Madurai India; 7 Department of Anatomy All India Institute of Medical Sciences-Bibinagar Bibinagar, Telangna India; 8 Department cum National Centre for Human Genome Studies and Research Punjab University Chandigarh India; 9 Department of Anatomy All India Institute of Medical Sciences-Jodhpur Jodhpur, Rajasthan India; 10 School of Allied Health Sciences (Nagpur) Datta Meghe Institute of Higher Education and Research Wardha, Maharashtra India; 11 Department of Anatomy All India Institute of Medical Sciences-Mangalagiri Mangalagiri, Andhra Pradesh India; 12 Department of Zoology Banaras Hindu University Varanasi, Uttar Pradesh India; 13 India Health Lead Oxford Policy Management Limited Oxford United Kingdom; 14 Department of Microbiology All India Institute of Medical Sciences-Bathinda Bathinda India

**Keywords:** SARS-CoV-2, COVID-19, epidemiology, demographic shift, severity of illness, variant, virus, pandemic, population studies, genomic analysis

## Abstract

**Background:**

The Delta variant (B.1.617.2) was considered the most dangerous SARS-CoV-2 strain; however, in-depth studies on its impact based on demographic and clinical characteristics of COVID-19 are scarce.

**Objective:**

We aimed to investigate the shift in demographic and clinical characteristics of the COVID-19 pandemic with the emergence of the SARS-CoV-2 Delta variant compared with the wild-type (WT) strain (B.1).

**Methods:**

A cross-sectional study of COVID-19 cases in the Indian population caused by the WT strain (B.1) and Delta variant of SARS-CoV-2 was performed. The viral genomic sequence metadata containing demographic, vaccination, and patient status details (N=9500, N_Delta_=6238, N_WT_=3262) were statistically analyzed.

**Results:**

With the Delta variant, in comparison with the WT strain, a higher proportion of young individuals (<20 years) were infected (0-9 years: Delta: 281/6238, 4.5% vs B.1: 75/3262, 2.3%; 10-19 years: Delta: 562/6238, 9% vs B.1: 229/3262, 7%; *P*<.001). The proportion of women contracting infection increased (Delta: 2557/6238, 41% vs B.1: 1174/3262, 36%; *P*<.001). However, it decreased for men (Delta: 3681/6238, 59% vs B.1: 2088/3262, 64%; *P*<.001). An increased proportion of the young population developed symptomatic illness and were hospitalized (Delta: 27/262, 10.3% vs B.1: 5/130, 3.8%; *P*=.02). Moreover, an increased proportion of the women (albeit not men) from the young (Delta: 37/262, 14.1% vs B.1: 4/130, 3.1%; *P*<.001) and adult (Delta: 197/262, 75.2% vs B.1: 72/130, 55.4%; *P*<.001) groups developed symptomatic illness and were hospitalized. The mean age of men and women who contracted infection (Delta: men=37.9, SD 17.2 years; women=36.6, SD 17.6 years; *P*<.001; B.1: men=39.6, SD 16.9 years; women=40.1, SD 17.4 years; *P*<.001) as well as developing symptoms or being hospitalized (Delta: men=39.6, SD 17.4 years; women=35.6, SD 16.9 years, *P*<.001; B.1: men=47, SD 18 years; women=49.5, SD 20.9 years, *P*<.001) were considerably lower with the Delta variant than the B.1 strain. The total mortality was about 1.8 times higher with the Delta variant than with the WT strain. With the Delta variant, compared with B.1, mortality decreased for men (Delta: 58/85, 68% vs B.1: 15/20, 75%; *P*<.001); in contrast, it increased for women (Delta: 27/85, 32% vs B.1: 5/20, 25%; *P*<.001). The odds of death increased with age, irrespective of sex (odds ratio 3.034, 95% CI 1.7-5.2, *P*<.001). Frequent postvaccination infections (24/6238) occurred with the Delta variant following complete doses.

**Conclusions:**

The increased involvement of young people and women, the lower mean age for illness, higher mortality, and frequent postvaccination infections were significant epidemiological concerns with the Delta variant.

## Introduction

The Delta variant (B.1.617.2) of SARS-CoV-2 caused COVID-19 waves and spikes in 2021 in multiple countries [1]. Structural and functional analyses of the lineage characterizing mutations in the spike glycoprotein have predicted potential alterations in virus-host interactions and masking of the antibody binding sites, leading to increased transmissibility, lethality, and immune escape capabilities for this variant [2-4], which were further validated in recent animal models [5,6] and human studies [7-10]. Available studies precisely indicate that the Delta variant is at least 50% to 60% more transmissible than the Alpha variant (B.1.1.7) [11] and is capable of immune escape against natural infections with previous SARS-CoV-2 strains, COVID-19 vaccines, and therapeutically used monoclonal antibodies [5-11].

The emerging SARS-CoV-2 variants reportedly vary in demographic characteristics, such as age- and sex-based vulnerability for contracting infection, developing severe illness, and mortality risk from wild-type (WT) strains (Wuhan strain and B.1) [4,12-15]. Against the established pattern of higher susceptibility for older adults and men, which is explained by documented immunological reasons [16-20], the emerging variants involve increasing proportions of the young and female sex [4,12-15]. However, studies that have examined the shift in demographic and clinical characteristics for the Delta variant are currently limited. A devastating second COVID-19 wave occurred in India in 2021, driven by the Delta variant [11,21]. An analysis of the official epidemiological data released by the government of India suggested [21] that a rise in new cases was recorded by February 2021; however, the rate of increase of new cases was slow until the beginning of March and was not reflective of an imminent second wave. A distinct new wave was not in view before the end of March when a sudden spurt was recorded in new cases. The rise in new cases then continued throughout April and peaked by the end of the first week of May 2021, when all global records were surpassed, reporting more than 0.4 million new cases daily. As early as April 2021, nearly 7 million new COVID-19 cases were recorded from the 19 million cases recorded in India during the pandemic. In addition, more than 48,000 deaths occurred in April, of 0.2 million deaths due to COVID-19 reported at that point in India [22]. A sudden rise in cases nationwide [21] shocked the health response system. The first COVID-19 wave, as in other parts of the world, was driven by WT strains in India [21]. The first variant with significantly increased transmissibility and lethality was the Alpha variant, which turned out to be a dominant strain by 2020 [21]. The Alpha variant caused a frequent increase in daily cases across the Indian states. In between, multiple other variants were also reported; however, none of those variants were able to contribute to a significant COVID-19 wave until the arrival of the Delta variant [21]. The first case of the Delta variant was reported in India by December 2020 [21]; however, an exclusive rise in Delta cases was only evident by the end of March 2021 [21]. The Delta variant–driven second wave continued for months, only seeing a decline in July 2021 [21].

As per the official estimates in September 2022, more than 3.4 million cases and 0.52 million deaths were recorded in India, and more than 89 million COVID-19 tests (632,930 per million population) and 200 million vaccinations (at least a single dose) were recorded in India [23]. Notably, the second wave contributed significantly to the total cases and deaths [21].

Plausibly higher transmissibility and virulence of the Delta variant and a lack of substantial vaccination at the time of the second wave could have been the likely reasons for the massive surge in new cases [4,11,21].

This study analyzed the shift in demographic and clinical characteristics of the COVID-19 pandemic in the Indian population with the emergence of the SARS-CoV-2 Delta variant compared with the WT strain (B.1).

## Methods

### Data Collection and Processing

A cross-sectional study was conducted to study the critical differences in demographic and clinical characteristics of the SARS-CoV-2 B.1.617.2 (Delta) variant infections compared with those caused by the WT B.1 strain (Wuhan strain with the D614G mutation) in the Indian population. Genomic sequencing of the virus strain isolated from patient samples is a standard method for confirmatory diagnosis of COVID-19 and identification of the causative SARS-CoV-2 variant. We analyzed the patient metadata information attached to the genomic sequence reports for the SARS-CoV-2 Delta and WT (B.1) strains detected in the patient samples from the Indian population. The SARS-CoV-2 genomic sequence reports from India (patient sample collection date no later than July 31, 2021) were accessed from the EpiCoV database of the Global Initiative on Sharing All Influenza Data (GISAID) [24] using the automatic search function for information by geographical location, SARS-CoV-2 lineage, and collection dates. A similar search strategy was used to retrieve comparative metadata for the COVID-19 cases with the WT B.1 strain reported on GISAID since the first COVID-19 wave. The metadata files of the Delta variant (test group) and WT (control group) strain sequences were downloaded from the individual GISAID accession numbers, checked and confirmed for accuracy of the lineage information, and screened for demographic details (age and sex) and vaccination status (for 2 complete doses). Further, the clinical outcomes (information about the illness severity and mortality during the disease course) of the WT and Delta variant strains cases were assessed using a repeat search entering “patient status” as additional input. The collected data were filtered by discarding the sequence reports containing no or incomplete information and duplications (repeat sequencing from the same individuals). To prevent selection bias in the data collection, no specific filters for age, sex, date of collection, and geographical location were used during the search. A team of 3 investigators rechecked and verified the sampling and data entry errors of the complete data set by matching the original details in the metadata files.

The sample size for the study was guided by serosurvey reports from the Indian population during successive waves of the pandemic [25,26]. Of note, nationwide seroprevalences of 24.1% and 67.6% were reported in the studies conducted at the end of the first and second waves, respectively [25,26]. Considering a minimum of 24.1% seroprevalence in the current population of India (approximately 1.35 billion) at 5% relative precision (1.25% absolute precision) and 95% CI, a minimum sample size of 4866 was determined for the study.

### Data Analysis and Presentation

#### Quantitative Variables

The final data were entered in Excel sheets, and the distribution was analyzed by age and sex. The age distribution of the Delta variant infections was presented at 10-year intervals. Additionally, for the analysis of clinical outcomes, all cases were divided into 3 broad age groups as follows: “young” (0-19 years), “adult” (20-59 years), and “older adult” (≥60 years).

#### Clinical Outcomes

Clinical outcomes of the positive cases were categorized as “asymptomatic/mildly symptomatic” (including cases in home isolation and/or quarantine with no overt symptoms or mild symptoms), “symptomatic/hospitalized” (including cases with overt symptoms, currently hospitalized, or released or recovered after hospitalization), and “demised.” These were also the significant outcomes assessed in this study.

#### Data Distribution and Analysis, Statistical Assessment, and Graphical Presentation

Individual outcomes were analyzed by age and sex distribution. To determine mortality, the statuses of all the categories other than “demised” were considered “living.” Categorical data are presented as frequency or proportion. Continuous data are presented as mean (or median) and SD. The relative contribution of the geographical locations within India (states and union territories) in the total available genomic data from India in the study period was noted; however, no further distinction of specific geographical locations was made while analyzing the final data.

Statistical tests were performed to evaluate intergroup differences in clinical outcomes with the help of Microsoft Excel 2019 and the XLSTAT package. The normality of the data was examined using the Kolmogorov-Smirnov test. For normally distributed data, 2-sample Student *t* tests and ANOVAs were used. The Games-Howell post hoc test was applied for intergroup comparisons. The Kruskal Wallis H test was used to analyze skewed data. The chi-square test was used for categorical variables. Multinomial logistic regression analysis was used to estimate the relative measures of effect, considering “demised” and “symptomatic/hospitalized” as the clinical outcomes in reference to the Delta variant versus the WT (B.1) strain. No specific theoretical nor statistical criteria were adopted for “age”; however, “B.1” for the virus strain and “men” for sex were set as the baseline or reference in the regression model.

Results were considered statistically significant at a *P* value ≤.05. Graphs were plotted to visualize the data trends.

### Ethical Considerations

Approval from the institute ethics committee was precluded, as the study used open-access data from the EpiCoVTM database from the GISAID [24]. GISAID permits open access to data to all individuals who agree to identify themselves and follow the GISAID sharing mechanism governed by its Database Access Agreement [27].

## Results

### Incidence

We assessed the genomic sequence reports for 8269 and 3767 cases with the Delta variant and WT B.1 strains, respectively, that were uploaded on GISAID from the first case to July 31, 2021. The SARS-CoV-2 genomic sequences were noted from nearly all the Indian states and union territories (Figure S1 in Multimedia Appendix 1). After filtering the data, 6238 Delta variant and 3262 WT B.1 strain cases were available for demographic analysis.

Among the screened cases, there were 24 Delta infections following 2 complete vaccine doses; however, no postvaccination infections were noted with B.1. In the database, 659 and 320 sequence reports with information on “patient status” were present for Delta and B.1 strains, respectively, and after filtering the data, 647 and 276 cases, respectively, were available for analysis regarding illness severity and mortality in terms of age and sex.

### Demographic Distribution

**Figure 1:**
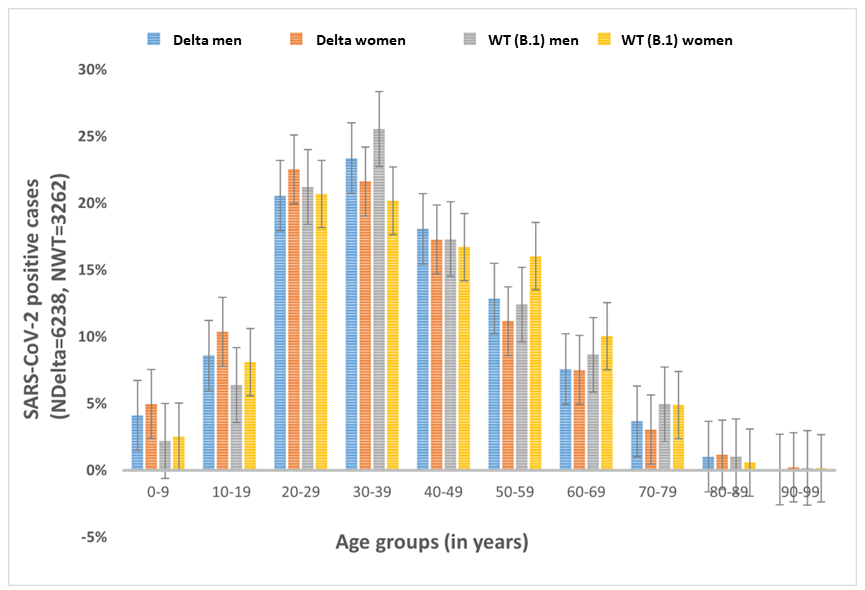
Demographic distribution of SARS-CoV-2 B.1.617.2 (Delta) variant versus the wild type (WT) strain (B.1) infections in Indian population.

Frequent incidences of Delta variant as well as B.1 strain infections (N=6238) were noted across the age groups, including in the young group (0-19 years; Figure 1). The prevalence of infections was higher with the Delta variant than with the B.1 strain in the young group (0-19 years), with the highest difference in prevalence in those aged 0 years to 9 years followed by those aged 10 years to 19 years (*P*<.001). Conversely, the differences in prevalences were relatively low in those aged 20 years to 60 years, with a few exceptions (*P*<.001; Figure 1). Although a greater proportion of men were infected with both variants (both sexes: N_Delta_=6238; N_WT_ =3262) than women (Delta: 3681/6238, 59% vs 2557/6238, 41%; WT: 2088/3262, 64% vs 1174/3262, 36%), the proportion of women contracting infection was higher with Delta than with B.1 (Delta: 2557/6238, 41% vs B.1: 1174/3262, 36%; *P*<.001). The mean ages when infected with the Delta variant were 37.9 (SD 17.2) years for men and 36.6 (SD 17.6) years for women and when infected with the B.1 strain were 39.6 (SD 16.9) years for men and 40.1 (SD 17.4) years for women (*P*<.001).

### Patient Status

The cases were noted for all categories for the Delta variant as well as B.1 (asymptomatic/mildly symptomatic: N_Delta_=72, N_WT_=24; symptomatic/hospitalized: N_Delta_=262, N_WT_=130; living but symptom status not known: N_Delta_=228, N_WT_=102; demised: N_Delta_ =85, N_WT_=20). The demographic (age groups and sex) distributions by patient status are shown in Figure S2 in Multimedia Appendix 1.

#### Symptomatic Illness and Hospitalization

**Figure 2:**
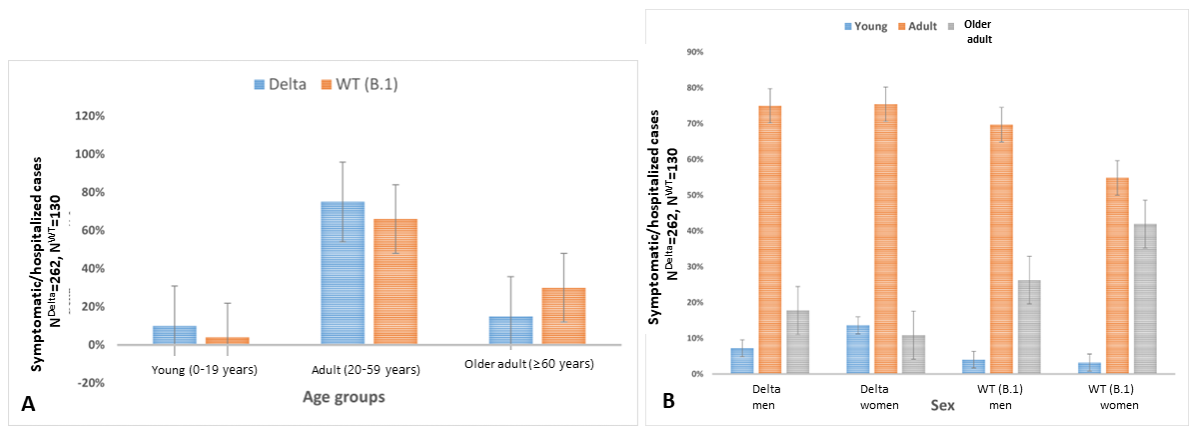
Distribution of symptomatic or hospitalized cases with SARS-CoV-2 B.1.617.2 (Delta) variant or wild-type (WT) strain (B.1) infections by (A) age (*P*=.02; statistical significance set at *P*<.05) and (B) sex (*P*<.001; statistical significance set at *P*<.001).

The cases who developed symptoms and required hospitalization were reported in each age group, including the young group (0-19 years) for both strains (Figure 2A). However, the young group (0-19 years; Delta: 27/262, 10.3% vs B.1: 5/130, 3.8%; *P*=.02) contributed to the increased proportion of the total number of symptomatic or hospitalized cases with the Delta variant. Further, higher proportions of young (Delta: 37/262, 14.1% vs B.1: 4/130, 3.1%) and adult (Delta: 197/262, 75.2% vs B.1: 72/130, 55.4%) women, but not men, developed symptoms and required hospitalization with the Delta variant than with B.1 (*P*<.001; Figure 2B). The mean ages at developing symptoms or hospitalization for men and women were 39.6 (SD 17.4) years and 35.6 (SD 16.9) years, respectively, with the Delta variant and 47 (SD 18) years and 49.5 (SD 20.9) years, respectively, with the B.1 strain (*P*<.001). However, in the multinomial logistic regression model, the risk of symptomatic illness or hospitalization was marginally higher for those of a lower age, with borderline statistical significance (odds ratio [OR] –1.1992, 95% CI 0.95-0.99; *P*=.046) for both strains. The viral lineage (OR 0.695, 95% CI 0.415-1.166; *P*=.17) and sex (OR 1.052, 95% CI 0.659-1.679; *P*=.83) had no significant effect on the estimates (Table 1).

**Table 1:** The viral and host factors influencing the clinical outcomes of death and symptomatic illness or hospitalization.

Clinical outcome and predictors			*z* distribution		*P* value		Odds ratio (95% CI)
**Death**							
	SARS-CoV-2 lineage: B.1^a^-Delta	3.93		<.001		3.034 (1.744-5.277)	
	Age	–9.10		<.001		0.934 (0.920-0.947)	
	Sex: men^a^-women	–1.11		.27		0.765 (0.477-1.227)	
**Symptomatic illness or hospitalized**							
	SARS-CoV-2 lineage: B.1^a^-Delta	–1.378		.17		0.695 (0.415-1.166)	
	Age	–1.992		.046		0.977 (0.956-0.999)	
	Sex: men^a^-women	0.213		.83		1.052 (0.659-1.679)	

^a^Reference predictors: SARS-CoV-2 lineage, age, and sex.

#### Total Mortality and Odds of Death

**Figure 3:**
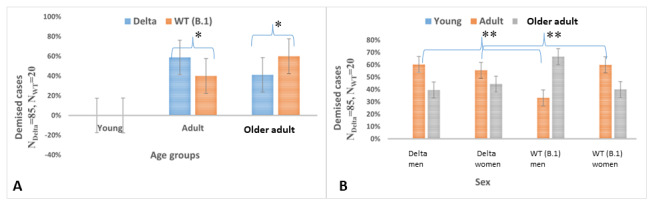
Distribution of demised cases with SARS-CoV-2 B.1.617.2 (Delta) variant versus wild type (WT) strain (B.1) infections by (A) age and (B) sex. *Statistical significance set at *P*<.05; **statistical significance set at *P*<.001.

Of the total cases, approximately 13.1% (85/647) and 7.2% (20/276) died due to COVID-19 caused by the Delta and B.1 strains, respectively. However, no deaths were reported for the Delta variant nor B.1 strain in those aged <20 years (Figure 3A). Further, the adult group contributed to a higher mortality with Delta variant infections than with B.1 strain infections (50/85, 59% vs 8/20, 40%; *P*=.01). When the adult and older adult age groups were analyzed together, there was greater mortality for men than women with the Delta variant (men: 58/85, 68% vs women: 27/85, 32%) and B.1 strain (men: 15/20, 75% vs women: 5/20, 25%; *P*<.001; Figure 3B). Notably, mortality with the Delta variant was lower for men (Delta: 58/85, 68% vs B.1: 15/20, 75%) but higher for women (Delta: 27/85, 32% vs B.1: 5/20, 25%) than with the B.1 strain (*P*<.001). The mean ages at illness-related mortality for men and women were 56.6 (SD 13.5) years and 58.8 (SD 11.4) years, respectively, with the Delta variant and 60.7 (SD 15.5) years and 50.2 (SD 15.7) years, respectively, with the B.1 strain (*P*<.001). The odds of death were higher with Delta variant infections irrespective of sex (OR 3.034, 95% CI 1.7-5.2; *P*<.001). Age but not sex influenced the effect estimates. Lower age was marginally protective (OR 0.934, 95% CI 0.92-0.95; *P*<.001) with both strains (Table 1).

### Postvaccination Infections

**Figure 4:**
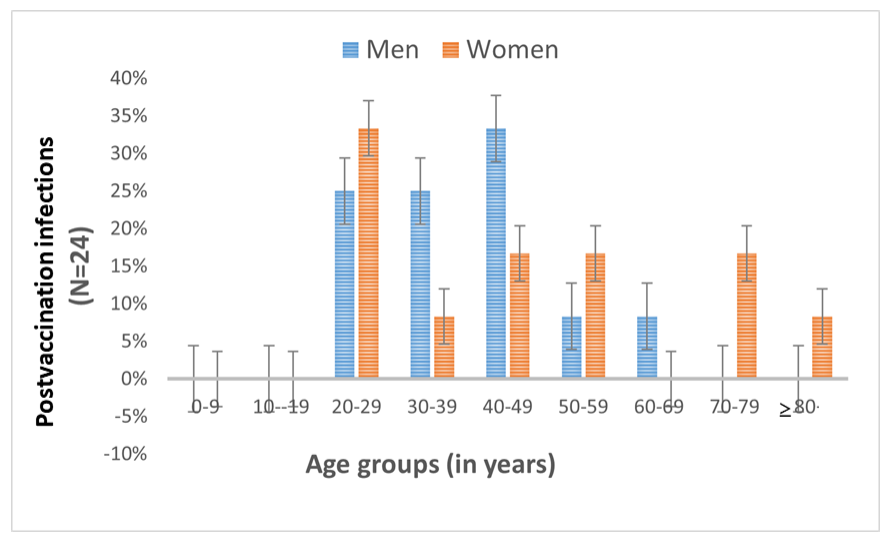
Demographic distribution of postvaccination (2 complete doses) SARS-CoV-2 B.1.617.2 (Delta) variant infections.

Of all the screened cases with the Delta variant or B.1 strain, Delta infections following 2 complete doses of vaccines did occur (24/6238: men=12, women=12); however, no postvaccination infections were noted with B.1 (0/3262). Postvaccination infections with the Delta variant were reported across the age groups (except for those aged 0-9 years or 10-19 years, which is explained by the fact that COVID-19 vaccines were not administered to individuals younger than 18 years in India until the period of this study) and for both sexes (Figure 4). The mean ages at postvaccination infection were 44.48 (SD 16.17) years for all cases, 40.4 (SD 11.9) years for men, and 47.8 (SD 20.4) years for women. No significant differences were noted in the frequency of postvaccination infections in terms of age groups and sex. Of all postvaccination infections, 88% (21/24) were with Covishield [28], and the rest (3/24, 12%) were with Covaxin [29].

## Discussion

### Principal Findings

Our analyses revealed essential observations indicating a shift in clinical and epidemiological characteristics of the COVID-19 pandemic from the WT strain B.1 to the Delta variant. With the Delta variant, more young individuals (<20 years) and adult women contracted the infection. In addition, an increased proportion of the young (<20 years) and adult population developed symptoms and required hospitalization. The mean age at COVID-19 infection and developing symptomatic illness or hospitalization was considerably lower. A disproportionate number of cases who developed symptomatic illness or were hospitalized was noted by sex: An increased proportion of young and adult women was affected. The mortality rate was about 1.8 times higher. Sex-specific contributions to the mortality data were lower for men but higher for women. However, the odds of death increased with age, irrespective of sex. Further, we noted multiple COVID-19 infections with the Delta variant following 2 complete vaccine doses.

Studies analyzing the demographic characteristics of the Delta variant in the Indian population are currently scarce. Recently, Kumar et al [30] presented their observations on the Indian population using data from the national clinical registry for COVID-19. The authors studied the clinical profiles of hospitalized patients during the second COVID-19 wave, mainly driven by the Delta variant [11,21]. Authors have also studied hospitalized cases in the first COVID-19 wave, primarily caused by WT strains [11,21]. However, the diagnosis of COVID-19 in cases included in this study was based on polymerase chain reaction test results, and no viral genomic sequencing was performed to ensure that the analyzed cases only represented those caused by the Delta variant or WT strains in the respective waves. The authors noted a decrease in the mean age of the total population who contracted infection and a reduction in the proportion of cases who were men during the second wave. The authors noted that mortality among hospitalized patients was 13.26% in the second wave, which was 3.1% higher than in the first wave. The increase in mortality was seen in all age groups in their study except for those aged <20 years, for whom mortality decreased [30]. We observed a similar trend in the susceptibility for contracting infection and risk of mortality in terms of age and sex, except we noted greater total mortality with the Delta variant (13.1%) than with the WT strain (7.2%). However, no deaths with either strain were noted in those aged <20 years in this study.

India is one of the world’s most populated countries, with a very high proportion of young people (approximately 45% are <20 years old) [31]. Our analyses show that, in comparison with B.1, Delta variant infections have occurred at considerably higher proportions in those <20 years old, particularly for those aged 0 years to 9 years (Delta: 4.5% vs B.1: 2.3%). The proportion of symptomatic cases and those requiring hospital admission was also higher in the young group (Delta: 10% vs B.1: 4%; Figure 2A). These findings indicate an age shift for the Delta variant, as COVID-19 infections with the WT strains were not common in young people and development of severe symptoms and the need for hospitalization were primarily limited to adults, more specifically older adults [17,18].

We observed that a higher proportion of women than men contracted the infection and developed symptoms or were hospitalized; in addition, mortality was lower in men but higher in women with the Delta variant than with the B.1 strain. Biologically, the severity of illness and mortality risks are considered lower for women than men for infectious diseases that affect mammals, including humans [32]. A higher vulnerability for contracting infection and symptomatic disease for women and narrowing the sex gap in mortality risk with the Delta variant present a paramount epidemiological concern.

Increased involvement of the younger age groups, primarily those aged 0 years to 19 years and of the female sex, in Delta variant infections compared with WT strains was indicated by studies and surveillance reports in the United Kingdom following a surge in Delta variant infections [8,33]. Despite the established immunological advantage for younger individuals [19,20,34] and women [16], as were reflected in infections with WT strains [16,18], increased risks of contracting infection and greater illness severity in these demographic groups indicate enhanced virulence and lethality with the Delta variant.

Last, the multiple Delta variant infections following 2 complete doses of COVID-19 vaccines in our study are supported by previous reports of vaccine breakthrough infections with this variant, including those from the Indian population [9,35].

The findings of this study and others describing demographic and clinical involvement in Delta variant infections confirm a shift from the established pattern for WT strains. Multiple factors could contribute to this epidemiological shift, most importantly, the variant’s intrinsic properties, such as increased transmissibility, virulence, and immune escape capabilities, as have been indicated by recent studies [7-9,11,30,36]. A sudden spurt in the number of cases during the second wave and consequent burden on the emergency health response system could have contributed to the increased mortality noted with the Delta variant. In contrast, the prioritized vaccination of those aged >45 years might have protected the vaccinated older adult population and contributed to lowering the mean age for COVID-19 involvement. On the other hand, in the initial period of the first COVID-19 wave, there was a shortage of diagnostic and COVID-19 care facilities and skilled health personnel in the country as well as limited knowledge about the prevention and therapeutic management of the pandemic. When combined, these limitations might have had an impact on the quality of the available epidemiological and clinical data related to WT strains.

### Limitations of the Study

Our study has multiple limitations that must be considered when interpreting the findings. First, our data for reporting postvaccination infections and patient status are limited; hence, related observations may require further validation from studies with a larger sample size. Second, the vaccination status data were unavailable in most of the accessed genomic sequence reports; hence, our data for postvaccination infections do not reflect the incidences of such cases in the total population. Our study does not rule out that actual incidences of postvaccination infections (after 2 complete doses) with the Delta variant could be higher. In addition, the noted 0 postvaccination infections with B.1 in our study could be an underestimation, as COVID-19 vaccines were yet to be made available to the masses in India during the first wave when the WT strains caused most of the infections.

Moreover, owing to the limited availability of vaccination status details for the screened cases in the studied database, we cannot comment conclusively on the efficacy of the vaccines noted in this study. Third, we have not considered the influence of confounding factors, such as comorbidities, vaccination status, and previous COVID-19 infection, on the clinical outcomes of the studied cases, which may have influenced the quality of the results. Last, although we applied no age, sex, nor region-specific filters when acquiring the data, a complete absence of selection bias cannot be assured when uploading the primary data on GISAID. However, any inherent bias in the primary data will likely be nondifferential and may not significantly impact the comparative analysis between the viral strains.

### Conclusions

In conclusion, our study reveals greater proportions of infected young individuals and women, a lower mean age for illness, greater mortality, and frequent postvaccination infections with the Delta variant than with the WT (B.1) strain. Collectively, the findings of this study elaborate on the changing demographic characteristics of the COVID-19 pandemic with the emergence of the Delta variant. These findings are essential because we have only considered in the analyses those COVID-19 cases for whom Delta variant infections were confirmed by genomic sequencing, ensuring the accuracy of the presented data.

## Appendix

Multimedia Appendix 1 Distribution of cases geographically and by patient status.

## Abbreviations

GISAID: Global Initiative on Sharing All Influenza Data
OR: odds ratio
WT: wild-type

## Data Availability

The primary data for this study are publicly available on the SARS-CoV-2 genomic sequence-GISAID database [24]. The tabulated data can be obtained from the corresponding author upon reasonable request.
