# Supplementary material for: Shift in Demographic Involvement and Clinical Characteristics of COVID-19 From Wild-Type SARS-CoV-2 to the Delta Variant in the Indian Population: In Silico Analysis
Source: Interact J Med Res. 2024 Oct 8;13:e44492. doi: 10.2196/44492 (PMC11496911; doi:10.2196/44492)
Supplement: Multimedia Appendix 1 [file ijmr_v13i1e44492_app1.docx]

**Figure S1. Geographical distribution of the studied cases with SARS-CoV-2 B.1.617.2 (Delta) variant and Wild type (WT) strain (B.1) infections across the Indian states and union territories (only the regions with more than 0.1% of the total cases for each SARS-CoV-2 strain have been shown in the pie chart).**

**
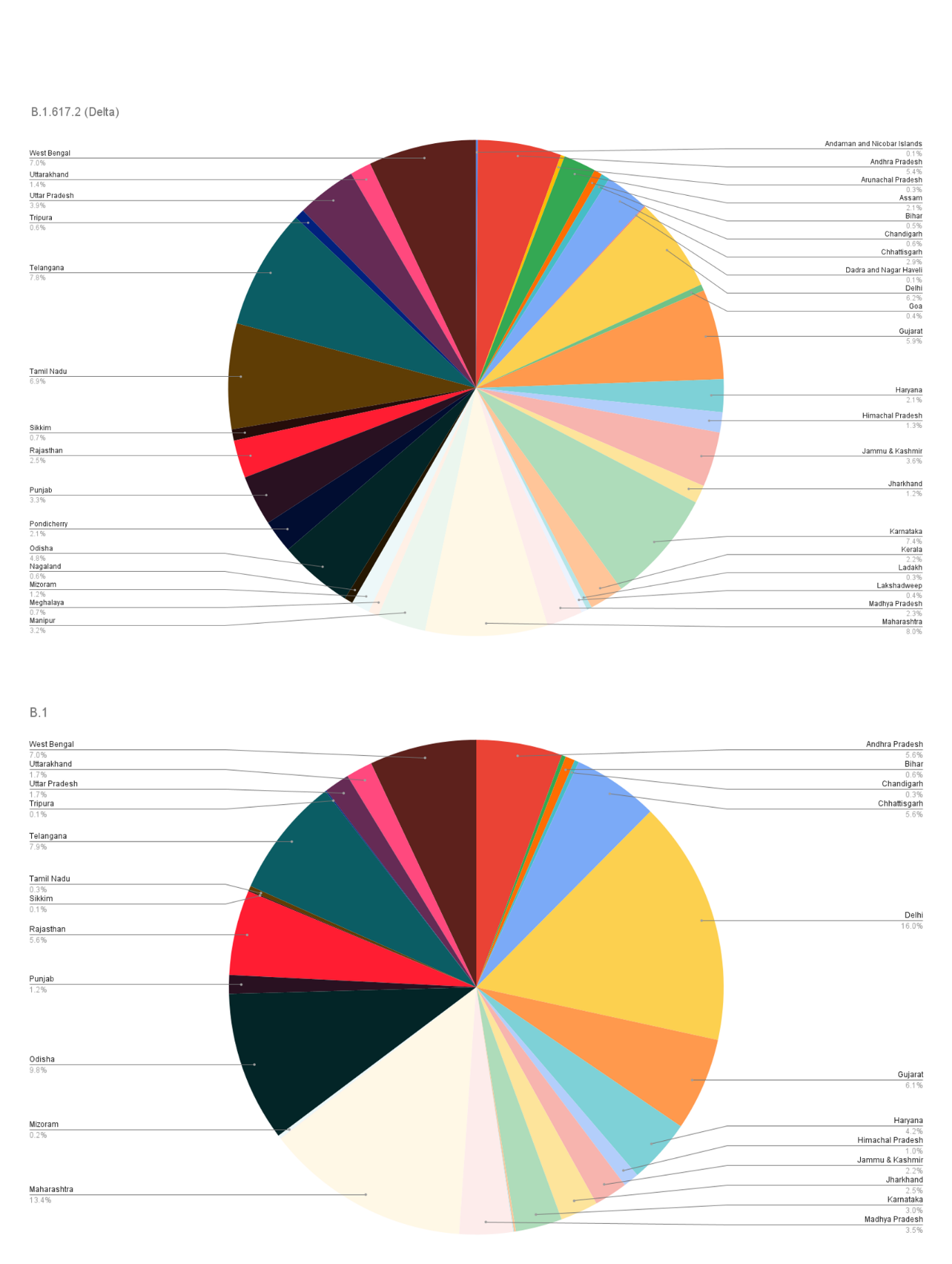
**

**Figure S2. Distribution of the cases with SARS-CoV-2 B.1.617.2 (Delta) variant and Wild type (WT) strain (B.1) infections in reference to patient status.**

**
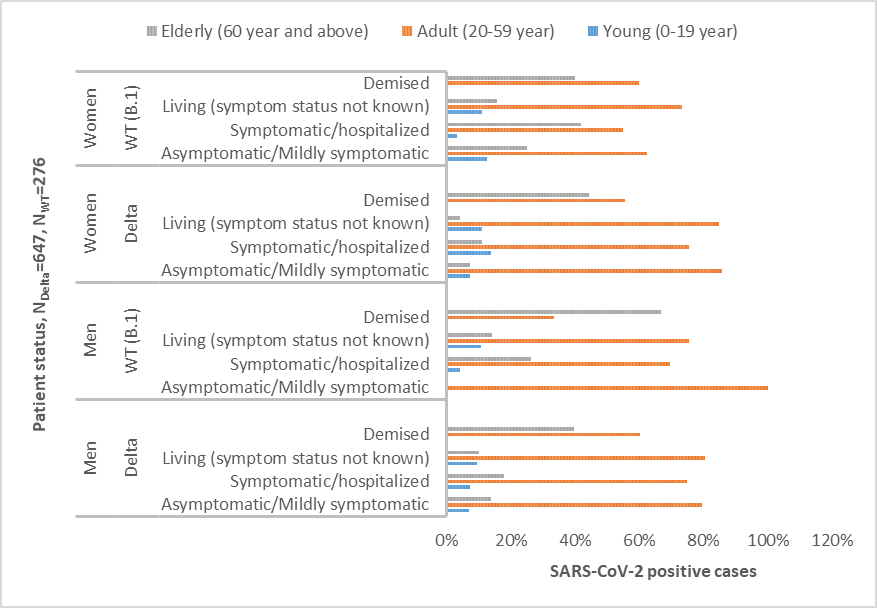
**
